# EBV Infection and Its Regulated Metabolic Reprogramming in Nasopharyngeal Tumorigenesis

**DOI:** 10.3389/fcimb.2022.935205

**Published:** 2022-07-01

**Authors:** Tingting Yang, Chanping You, Shuhui Meng, Zhengquan Lai, Weipeng Ai, Jun Zhang

**Affiliations:** ^1^Department of Pharmacy, Shenzhen University General Hospital, Shenzhen, China; ^2^Department of Pathology, Li Ka Shing Faculty of Medicine, The University of Hong Kong, Hong Kong, Hong Kong SAR, China; ^3^Clinical Medical Research Center, Guangdong Provincial Engineering Research Center of Autoimmune Disease Precision Medicine, Shenzhen Engineering Research Center of Autoimmune Disease, Shenzhen People’s Hospital, Shenzhen, China; ^4^Guangdong Key Laboratory of Genome Instability and Human Disease Prevention, Department of Biochemistry and Molecular Biology, Shenzhen University School of Medicine, Shenzhen, China

**Keywords:** EBV infection, metabolic reprogramming, nasopharyngeal carcinoma, NPC pathogenesis, therapeutic strategies

## Abstract

Viral oncogenes may drive cellular metabolic reprogramming to modulate the normal epithelia cell malignant transformation. Understanding the viral oncogene–mediated signaling transduction dysregulation that involves in metabolic reprogramming may provide new therapeutic targets for virus-associated cancer treatment. Latent EBV infection and expression of viral oncogenes, including latent membrane proteins 1 and 2 (LMP1/2), and EBV-encoded BamH I-A rightward transcripts (BART) microRNAs (miR-BARTs), have been demonstrated to play fundamental roles in altering host cell metabolism to support nasopharyngeal carcinoma (NPC) pathogenesis. Yet, how do EBV infection and its encoded oncogenes facilitated the metabolic shifting and their roles in NPC carcinogenesis remains unclear. In this review, we will focus on delineating how EBV infection and its encoded oncoproteins altered the metabolic reprograming of infected cells to support their malignances. Furthermore, based on the understanding of the host’s metabolic signaling alterations induced by EBV, we will provide a new perspective on the interplay between EBV infection and these metabolic pathways and offering a potential therapeutic intervention strategy in the treatment of EBV-associated malignant diseases.

## Epstein–Barr Virus and EBV Infection

Epstein–Barr virus (EBV) is the first human oncogenic virus discovered in 1964 and has been currently identified in more than 90% population all over the world (Epstein et al., 1964; Cohen et al., 2011). From being a γ-herpesviruses, EBV, also known as human herpesvirus type 4 (HHV4), composed the human pathogenic herpesvirus family together with three α-herpesviruses: herpes simplex virus 1 and 2 and also varicella zoster virus; three β-herpesviruses: cytomegalovirus and HHV 6 and 7; and one γ-herpesvirus: HHV8 (Whitley, 1996). The virus consists of an outer bilayer envelop, a protein tegument, and a nucleocapsid core (Li et al., 2020). The outer bilayer envelop is composed of virus-encoded glycoprotein that functions as host cell receptor recognition, cell tropism, and cell membrane fusion. The nucleocapsid containing 162 capsomers is located at the center of the virus, where it enveloped a linear double-strand DNA with a size of 168–184 kbp. The EBV DNA genome encodes approximately 85 genes, these including but not limiting to genes in contributing to EBV infection, replication, and metabolic reprogramming (Zhang et al., 1018; Hammerschmidt and Sugden, 2013; Kang and Kieff, 2015; Piccaluga et al., 2018). In primary infection, EBV usually does not cause any symptom. The virus is believed to enter the human body through the Waldeyer’s tonsillar ring, which surrounds the nasopharynx and oropharynx. EBV may be transmitted to the mucosal surface of the ring through saliva, crossing or infecting the epithelial cells to further infect the underlying B lymphocytes (Duca et al., 2007; Thorley-Lawson et al., 2013). The cluster of differentiation 21 (CD21; also known as CR2), a major receptor for EBV infection, provides a barrier-free path for the virus to enter B cells (Hutt-Fletcher, 2007). Due to the lack of CD21 in epithelial cells, EBV infected epithelial cell by different means of mechanism, which may through fusing the viral envelope and epithelial cell membrane by the interaction of EBV-encoded gL/gH and the cell-expressed integrin αvβ5/6/8 to release the virions into the cell, entering the cell directly with/without the association with the viral-encoded BMRF2 and α5β1 and through other viral and cellular molecular interactions (Tsang et al., 2014).

EBV can undergo lytic replication or latent infection depending on different circumstances (McKenzie and El-Guindy, 2015). In the EBV latently infected memory B cells, differentiation of the cells into plasma cells will reactivate the lytic program to produce tremendous infectious virions, thereby to increase the possibility of new host cells infection (Hatton et al., 2014). In normal epithelial cells, the default mode of EBV infection is lytic infection, although there are increasing evidence indicating that the lytic infection of EBV may also contribute to oncogenesis in epithelial cancers (Rosemarie and Sugden, 2020; Frappier, 2021), including EBV-associated gastric carcinoma (EBVaGC) and nasopharyngeal carcinoma (NPC), EBV persists predominantly in a latency manner (Tsao et al., 2017). Thus, the successful establishment of latent infection by EBV in host cells has long been postulated to be an essential hallmark for malignant transformation. During the latent stage of EBV, only limit genes were expressed, and these genes are named latent genes. They were latent membrane proteins (*LMPs*), EBV-encoded nuclear antigens (*EBNAs*), BamH1-A region rightward transcript (*BARTs*) RNAs, and EBV-encoded small RNAs (*EBERs*), some of which have been reported to be included in the oncogenic role of EBV (Zhang et al., 1018; Zhu et al., 2016a; Tsao et al., 2017; Zhu et al., 2022).

## EBV Infection in Tumorigenesis

As a group I carcinogen, EBV causes around 2% of all cancer cases worldwide every year, typically including NPC, EBV-associated lymphomas, and EBVaGC (Young and Dawson, 2014; Yin et al., 2019; He et al., 2022). Recent study has also identified that long-term EBV infection results in chronic inflammatory demyelinating disease of the central nervous system, such as multiple sclerosis (Bjornevik et al., 2022; Bordon, 2022; Robinson and Steinman, 2022; Sollid, 2022). *In vitro* data have indicated that the EBV-infected B lymphocytes will be transformed into continuous proliferative cells (Yin et al., 2019). In immunocompetent individuals, the infection of EBV triggers the cytotoxic immune response of the host to against the virus, and, finally, the number of EBV-infected cells is maintained in a homeostatic level. In immunocompromised people, like AIDS patients and immunosuppressants recipients, however, EBV-infected cells may escape from effective T-cell surveillance and become uncontrolled in cell proliferation, which ultimately leads to cancers (Young and Rickinson, 2004).

NPC is the closest EBV-associated endemic disease, in which has higher prevalence in Africa and Southeast Asia, but lower incidence in western countries. According to the latest tumor classification by the World Health Organization (WHO) (5^th^ edition), NPC can be divided into three categories: keratinizing squamous cell carcinoma (type I), non-keratinizing squamous cell carcinoma (type II), and basaloid squamous cell carcinoma (type III) (Badoual, 2022). Non-keratinizing NPC is predominantly associated with EBV infection, of which nearly 100% of the undifferentiated and partially differentiated cases are detected as EBV-positive (Young and Dawson, 2014). Despite that the role of EBV in the etiology of NPC remains mysterious, the viral infection has been long considered as one of the major risk factors that contributes to the development of NPC (Tsao et al., 2014). Classical Hodgkin’s lymphoma (cHL) and Burkitt’s lymphoma (BL) are two typical EBV-associated B-cell lymphomas. In cHL, it is observed that approximately 40% of the cases are EBV-positive in developed countries, and this ratio will be even higher in developing counties (Vrzalikova et al., 2021). In BL, endemic BL in Equatorial Africa and Papua New Guinea especially in the regions with high incidence of malaria, demonstrates a strong correlation with EBV, in which the virus can be detected over 95% of all the cases (Brady et al., 2007; Hutcheson et al., 2020). While in sporadic BL (sBL), which is a rare form of BL but more frequently prevalent in western countries, EBV-positive tumors are only account for 20%–30% of total cases (Pannone et al., 2014; Satou et al., 2015). Unsurprisingly, EBV infection has been implicated to be one of the risk factors in HIV-associated cHL and BL, by means of statistical analysis to show that HIV-positive tumors were more likely to be EBV-positive cases (Glaser et al., 2003; Shindiapina et al., 2020). The infection of HIV may enhance the pathogenic capacity of EBV by lowering the immunity of the host. Furthermore, natural killer/T-cell lymphoma (NKTL) is another group of EBV-associated lymphoma that is commonly found in Asia and Latin America. The tumors derived from the transformed NK/T cells and it has been diagnosed that a number of NKTL are EBV-positive (Swerdlow et al., 2014). Healthy NK/T cells may be infected when the NK/T cells are in respond to EBV-infected cells. Nevertheless, the mechanism remains to be investigated. EBVaGC is a subset of gastric cancer family, which was reported more likely to be occurred in males and patients under the age of 60; however, it only accounts for around 9% of all the cases (Sun et al., 2020). Remarkably, from NPC and EBV-associated lymphomas to EBVaGC, an inescapable fact is that there is a considerable proportion of EBV-attributable tumors diagnosed as EBV-negative, whereby brings the “hit-and-run” hypothesis to the EBV pathogenesis. In this theory, the viral gene expression followed by the EBV infection, driving somatic mutations and activating the cellular oncogenes, played fundamental roles in driving EBV-associated malignances. Once the cells acquired a stable and heritable malignant feature, the virus may not be a critical factor for cell propagation; therefore, the transformed cells will steadily lose the EBV accompanied with the continuously proliferation (Ambinder, 2000; Mundo et al., 2020). However, there is only a limited direct evidence to support this idea.

Multiple mechanisms had been proposed to elucidate the roles of EBV in tumorigenesis. One of the assumptions is that EBV induces a series of glucose metabolic reprogramming events in infected cells, thereby driving healthy cells to transform to malignant cells. Standing on this point, studies focused on cell metabolic reprogramming in B-cell lymphomas have reported that EBV or EBV-encoded latent genes, like *LMP1*, *EBNA2*, *EBNA-LP*, and *EBNA-3A*, were able to immortalize and transform the cell *via* modulating multiple metabolic genes and enhancing the aerobic glycolytic pathway (Sommermann et al., 2011; Darekar et al., 2012; Pich et al., 2019; Wang et al., 2019). In this review, we focused on discussing the roles of EBV infection that reprogrammed metabolic shift in NPC tumorigenesis.

## EBV Infection Induces Metabolic Reprogramming in NPC

**Figure 1:**
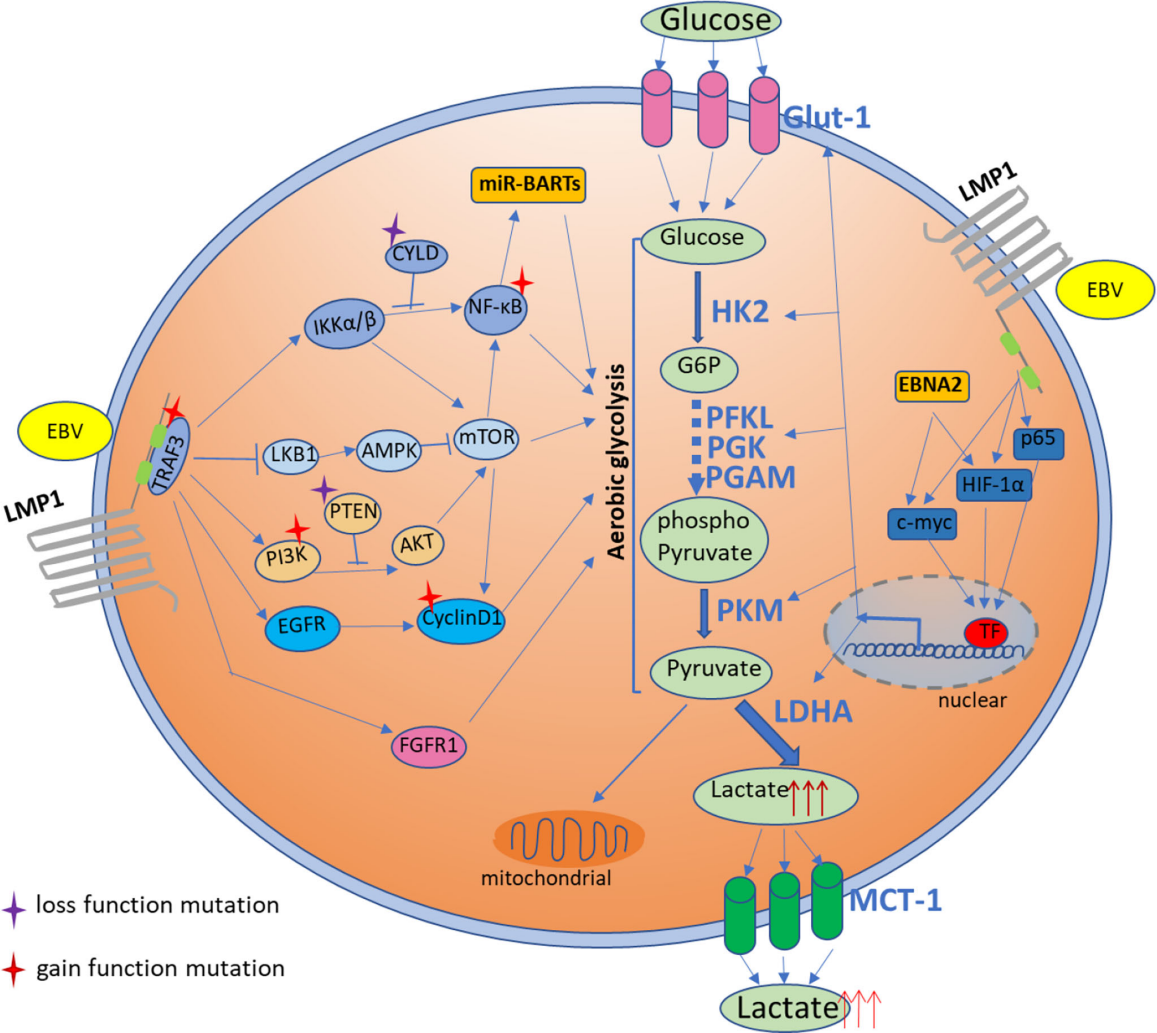
Activation of multiple signaling pathways that involved in glucose metabolism in NPC cells by EBV-encoded onco-products as well as the somatic-mutated genes identified in NPC. EBV-encoded latent gene product, LMP1, involves in regulation of glucose metabolism through multiple pathways including AMPK/mTOR, PI3K/AKT/mTOR, IKK/NF-κB, FGFR and cyclin D1 signaling. The common somatic mutations in NPC targeting to these multiple presented pathways are also shown to involve in glucose metabolism. EBV infection also regulates glucose metabolism through multiple transcription factors, like HIF-1α and c-Myc, that mediated glycolytic enzymes activation. EBV microRNA also involves in regulation of glucose metabolism in NPC.

EBV latently infection brings about viral-encoded latent genes to the host cell, some of which are observed to be able to regulate the glucose metabolism–related pathways to induce cellular metabolic alteration, further steadily transforming healthy cells into neoplastic cells **(** **Figure 1** and **Table 1****)**. Thus, EBV infection is believed to be involved in the initiation of NPC pathogenesis. Unfortunately, the detailed mechanism for EBV-associated tumorigenic process is still not fully understood.

**Table 1:** EBV-encoded oncogenes and targeted signaling and metabolic enzymes.

EBV-Encoded Oncogenes	Targeted Pathways or Cellular Process	Targeted Metabolic Enzymes	Ref.
LMP1	↓ HoxC8	↑ HK2 and ↑ Glut1	(Jiang et al., 2015)
	↑ DNMT1	↓ OXPHOS	(Luo et al., 2018)
	↓ GSK3β, ↑ PI3K/AKT and ↑ c-Myc	↑ HK2	(Xiao et al., 2014)
	↑ c-Myc	↑ IDH2	(Shi et al., 2020)
	↑ FGFR1 and ↑ FGF2	↑ HIF-1α, ↑ LDHA, ↑ PDHK1, ↑ PKM2 and ↑ PDHA1	(Lo et al., 2015)
	↓ TTP, ↓ PUM2, ↑ ERK1/2 and ↑ STAT3	↑ HIF-1α	(Sung et al., 2016)
	↓ CYLD and ↑ NF-κB	↑ PFKFB3	(Edwards et al., 2015)
	↑ mTORC1 and ↑ NF-κB	↑ Glut-1	(Zhang et al., 2017)
	↑ mTORC2	↑ PDHE1α	(Zhang et al., 2019)
LMP2A	↑ mTORC1	–	(Moody et al., 2005)
	↑ ECAR	–	(Zheng et al., 2020)
miR-BART4	↓ PTEN and ↑ PI3K/AKT	–	(Wu et al., 2018)
miR-BART7-3p	↑ PTEN/PI3K/Akt, ↑ c-Myc and ↑ c-Jun	–	(Cai et al., 2015a)
miR-BART8-3p	↑ NF-κB and ↑ ERK1/2	–	(Lin C et al., 2018)
miR-BART22	↑ PI3K/AKT	–	(Liu et al., 2019)
miR-BART1-5P	↓ PTEN, ↓ AMPKα1 and ↑ AMPK/mTOR/HIF1	↑ GLUT1, ↑ HK2 and ↑ LDHA	(Lyu et al., 2018)
miR-BART1	–	↑ G6PD, ↑ PHGDH, ↑ PAST1, ↑ IDH2, ↓ UGT8, ↓ LDHB, ↓ SGPL1 and ↓ DHRS3	(Ye et al., 2013)

"↑" means increased activity or expression level.

"↓" means decreased activity or expression level.

### EBV-Encoded Latent Membrane Proteins

LMP1, an oncoprotein encoded by EBV, has been well documented to contribute to the NPC metabolic reprogramming. LMP1 boosts the aerobic glycolysis by modulating several oncogenic signaling pathways. Specifically, LMP1 negatively regulated an aerobic glycolysis suppressor HoxC8 through RNA polymerase II (RNA Pol II) stalling to enhance the glycolytic pathway genes (Jiang et al., 2015). A study revealed that LMP1 can activate AKT and suppress the activity of oxidative phosphorylation (OXPHOS) by upregulating the expression level of DNA methyltransferases 1 (DNMT1) (Luo et al., 2018). Activation of PI3K/AKT by LMP1 decreased the activity of glycogen synthase 3β (GSK3β), which further stabilized the oncoprotein c-Myc. Since c-Myc is a hexokinase 2 (HK2) transcription factor, the action of LMP1 ultimately enhanced the signal of HK2 and thereby promoted the glycolysis process (Xiao et al., 2014). Furthermore, upregulation of c-Myc by LMP1 promoted the transcriptional activity of wild-type *IDH2*, which is a key enzyme involved in the mitochondrial metabolic process, leading to a significant metabolic change in NPC cells (Shi et al., 2020). LMP1 can also activate one of the PI3K/AKT upstream pathways, the fibroblast growth factor receptor 1 (FGFR1) pathway, by increasing the expression levels of both FGFR1 and its ligand fibroblast growth factor 2 (FGF2), consequently enhancing the glycolytic system by inducing the activities of hypoxia-induced factor 1 alpha (HIF-1α), lactate dehydrogenase (LDHA), and pyruvate dehydrogenase kinase 1 (PDHK1), but suppressing the activities of the pyruvate kinase M2 isoform (PKM2) and pyruvate dehydrogenase A1 (PDHA1) (Lo et al., 2015). Moreover, a direct regulation of LMP1 on HIF-1α was reported. LMP1 significantly upregulated protein and the mRNA level of *HIF-1α* by reducing the expression level of tristetraprolin (TTP) and pumilio RNA binding family member 2 (PUM2), which are two RNA-destabilizing proteins, to stabilize the *HIF-1α* RNA. The carboxy-terminal activating region 1 (CTAR1) and 3 (CTAR3) of LMP1 were involved in this program by which the former structure interacts with the extracellular signal–regulated kinase 1 and 2 (ERK1/2) pathway and the latter interacts with the STAT3 pathway. CTAR1 also played a role in the ERK1/2/NF-κB (nuclear factor κB) pathway to enhance the *HIF-1α* promoter activity (Sung et al., 2016). The activity of NF-κB signals has been reported to contribute to metabolic alteration (Mauro et al., 2011; Johnson and Perkins, 2012). In NPC, aberrant activation of NF-κB pathway, which may result from the inactivating mutations of NF-κB–negative regulators such as NFKBIA, CYLD, and TNFAIP3 (Zheng et al., 2016; Li et al., 2017), was universally observed and it is believed to be closely related to the infection of EBV (Lo et al., 2006; de Oliveira et al., 2010; Lin W et al., 2018; Tay et al., 2022). Consistently, EBV infection was showed to downregulate the expression levels of CYLD in NPC cells (Li et al., 2021), and CYLD deficiency could increase the transcriptional activity of *PFKFB3*, which is a key regulator of glycolysis, to enhance glycolysis (Wang et al., 2022). Furthermore, the CTAR1 and CTAR2 of LMP1 have also been demonstrated to distinctly activate NF-κB (Edwards et al., 2015). Our previous study demonstrated that LMP1 is responsible for the elevated transcriptional activity of *Glut-1*, resulting from the activation of mechanistic target of rapamycin complex 1 (mTORC1) and NF-κB pathways (Zhang et al., 2017). Accompanied by our deeper exploring, more recently, we further found that LMP1 can also enhance the glucose metabolic programming by activating a glucose metabolic enzyme PDHE1α, which is mediated by the mTORC2 signaling (Zhang et al., 2019). Apart from LMP1, another EBV-encoded latent membrane protein LMP2A was observed to be able to activate the mTORC1 signaling (Moody et al., 2005). A recently published paper suggested that the extracellular acidification rate (ECAR) was increased in the LMP2A-expressed NPC cell lines when compared to their parental cells, indicating an enhanced glycolysis activity induced by LMP2A (Zheng et al., 2020). Nevertheless, to specifically explain more the role of LMP2A in aerobic glycolysis, studies on investigating the detailed molecular mechanisms need to be proposed.

### EBV-Encoded BamH I-A Rightward Transcripts (microBARTs)

To discuss EBV-associated cancers, EBV-encoded microRNAs (miRNAs) are an inescapable member to be involved that is highly expressed in EBV-positive tumors and they are believed to contribute to the pathogenesis of EBV. There are more than 40 endogenous microRNAs (miRNAs) identified to date, the most abundant of which are EBV-encoded BamH I-A rightward transcripts (BARTs) in EBV-associated epithelial cancers, including NPC (Lo et al., 2012; Kuzembayeva et al., 2014). The underlying function of BART miRNAs in cancer development remains under investigation, particularly in recent years. Some of the BARTs, such as miR-BART3, miR-BART4, miR-BART7-3p, miR-BART8-3p, miR-BART19-3p, and miR-BART22, have been suggested to play the roles in enhancing cell proliferation, invasion, metastasis, and radio/chemo-drug resistance by different mechanisms in NPC (Lei et al., 2013; Cai et al., 2015a; Wu et al., 2018; Lin C et al., 2018; Liu et al., 2019; Zhang et al., 2020). Specifically, miR-BART4 was reported to downregulate the tumor suppressor phosphatase and tensin homolog (PTEN) thus facilitate the activation of PI3K/AKT and involved in metabolic regulation (Wu et al., 2018); miR-BART7-3p not only activate the PTEN/PI3K/Akt but also upregulate c-Myc and c-Jun (Cai et al., 2015a); miR-BART8-3p could excite both NF-κB and ERK1/2 pathways (Lin C et al., 2018); and miR-BART22 could also activate the PI3K/AKT pathway (Liu et al., 2019). It is worth noting that the pathways were activated by the miR-BARTs mentioned above also have been correlated to the improvement of the glycolysis activity. However, whether the presence of these EBV-miR-BARTs will eventually enhance the tumor aerobic glycolysis program needs to be further investigated. By contrast, direct evidence has indicated that another EBV-miR-BART, miR-BART1-5P, is able to promote cell glycolysis in terms of more lactate production, higher glucose uptake and assumption, and more ATP generation mechanistically by downregulating PTEN and the α1 catalytic subunit of adenosine 5´-monophosphate–activated protein kinase (AMPKα1) (Lyu et al., 2018). Downregulation of PTEN to activate its downstream pathways by miR-BART1 also has been reported in another study, further supporting the role of miR-BART1 as a PTEN suppressor (Cai et al., 2015b). Moreover, several metabolism-associated genes were also observed to be modulated in miR-BART1–overexpressed cells, in which miR-BART1-5P upregulated *GLUT1*, *HK2*, and *LDHA* (Lyu et al., 2018), miR-BART1 upregulated *G6PD*, *PHGDH*, *PAST1*, and *IDH2*, but downregulated *UGT8*, *LDHB*, *SGPL1*, and *DHRS3* (Ye et al., 2013). These studies revealed that miR-BART1 participated in the metabolic reprogramming through multiple pathways in NPC.

## EBV-Mediated Metabolic Reprogramming Contributes to the NPC Pathogenesis

**Figure 2:**
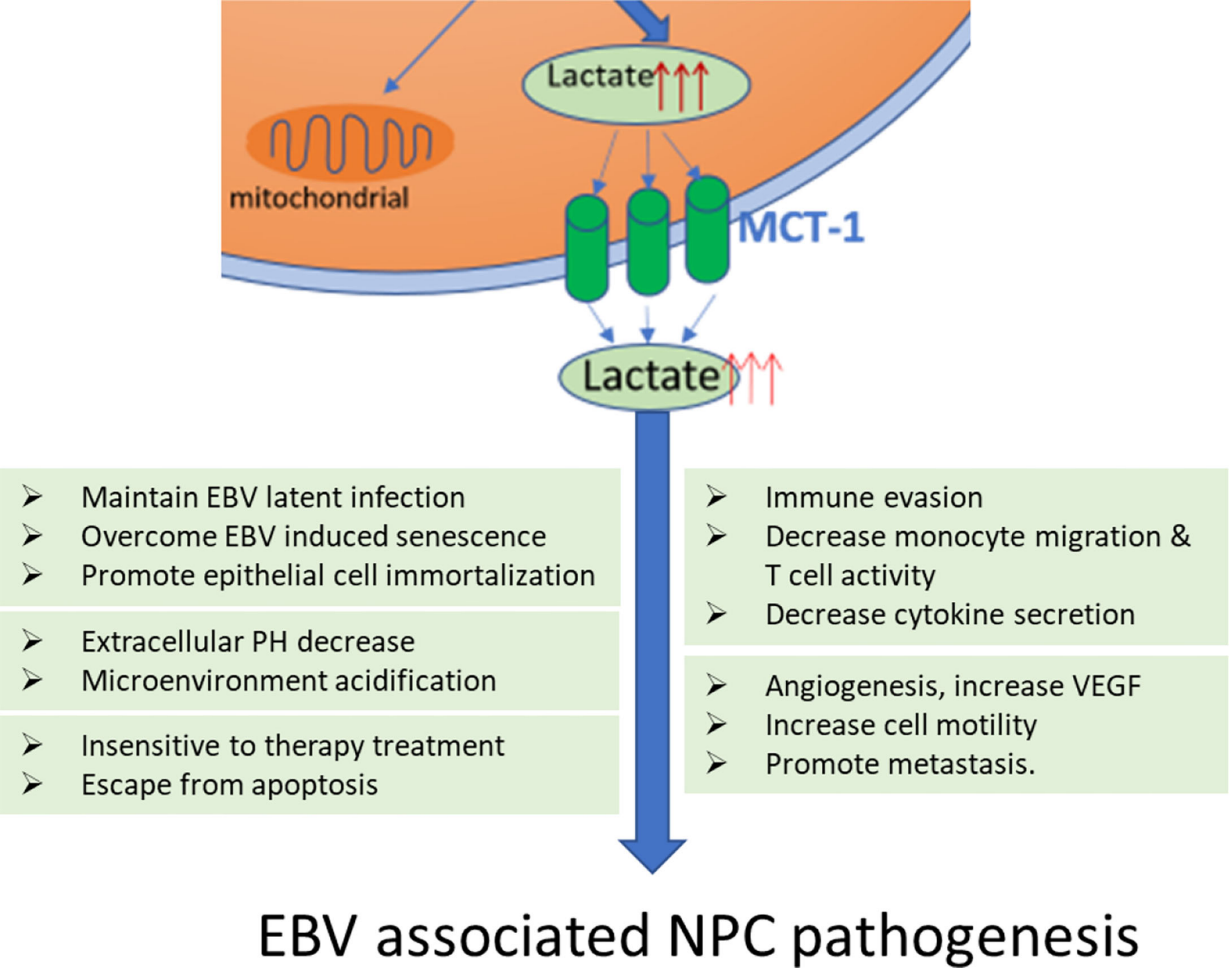
Lactate produced by EBV enhanced aerobic glycolysis contributes to all the major steps in NPC pathogenesis. Lactate increases the expression of vascular endothelial growth factor (VEGF) stimulating angiogenesis, increases extracellular acidosis of tumor microenvironment to beneath the cancer cell motility and metastasis. Lactate is also involved in the ‘immune escape’ by decreasing monocyte migration and decreased activation of T cells as well as cytokine release. Finally, lactate is necessary for protecting cancer cells escape from apoptosis and resistance to therapy.

Tumor cells generally use glucose for aerobic glycolysis, rather than metabolize glucose in the oxidative phosphorylation pathway, and this event was named the Warburg effect (Warburg, 1956). As one of the 10 hallmarks of cancer, aerobic glycolysis finally pushes the cancer cells into its infinite replicative potential, self-sufficiency in growth signals, and resistance to antigrowth signals; overcomes senescence and immortalization; escapes from apoptotic induction; and disturbs the microenvironment, angiogenesis, tissue invasion, and metastasis (Hanahan and Weinberg, 2011). As a herpesvirus, EBV and its encoded onco-products have the potential to alter glucose metabolism that has been discussed above, this event may contribute to NPC carcinogenesis through multiple approaches. Details of the potential involvements of glucose metabolism that contributes to carcinogenesis of EBV-associated NPC will be discussed below (**Figure 2**).

### Glucose Metabolism Contributes to EBV-Associated Immortalization

Immortalization is a prerequisite property of cancer cells and is regarded as an early conversion from premalignancy into malignance in human carcinogenesis (Hanahan and Weinberg, 2011). Experimentally, many factors can induce cell immortalization, such as viral infection and genetic alteration that led to abnormal cell proliferation, and conspire to confer replicative immortality can contribute to immortalization. Thus, defining and elucidating the molecular and cellular mechanisms of immortalization could provide important insights into the early stage of carcinogenesis. EBV infection has the property to immortalize B cells, contributing to human B-cell entry into an uncontrolled proliferation and further transformation (Brickell, 1992; Manor, 2008; McFadden et al., 2016). The metabolic stress is a barrier for EBV-mediated B-cell immortalization, and high-glucose metabolism would enhance the cell proliferation and immortalization (McFadden et al., 2016). Interestingly, during immortalization, several biological events that are preventing this process are emerged, and one of them is bypassed senescence. Normally, primary cells undergo senescence after a period of extended culture or exposed to reactive oxygen species inducible reagents (Shay and Roninson, 2004). Enhanced glycolysis could modulate cellular life span by preventing the primary cultures from oxidative stress or oncogene expression status; in other words, the enhanced glycolysis could help cells to overcome the senescence and contributes to growth and escape from apoptosis (Kondoh et al., 2007). This theory could explain why LMP1 expression in NPE cells could reverse the oncogene Ras-induced senescence and further accelerates cancer development by preventing premature senescence induced by mitogenic oncogenes (Yang et al., 2000). In nasopharyngeal epithelial cells, infection with EBV at an early stage occurred simultaneously with slowing down the cellular growth and accelerating senescence, overexpression of cyclin D1 or its associated factors in the NPE cell lines, however, could counteract the EBV infection induced arrest and senescence (Tsang et al., 2012); this may be due to the activation of cyclin D1 pathway that enhances the aerobic glycolysis property and further overcomes the senescence, therefore enabling persistent infection of EBV and drives NPC pathogenesis. We have reported that EGFR/mTOR/NF-κB axis activation supports the growth and highly proliferative of hTERT-immortalized NPE cells, as discussed above that NF-κB signaling activation is important for the development of NPC (Zhu et al., 2016a). Thus, the observation that NF-κB activation facilitates growth of immortalized NPE cells may represent an early stage of NPC pathogenesis. EBV-encoded oncoprotein, LMP1, is also reported to drive the life-span extension of primary cultures of NPE cells and finally facilitate its immortalization; in early stage of this case, the c-Myc, Bmi-1, and Id-1 were upregulated but p21 and p16 expression were downregulated in a late stage of immortalization (Yip et al., 2010). Because enhanced glucose metabolism is a typical and prominent feature of cancer cells, therefore, a well clarification on EBV infection-enhanced aerobic glycolysis that overcomes senescence and accelerates immortalization will provide important insights into understanding of NPC carcinogenesis.

### Glucose Metabolism Contributes to EBV Latency Infection

EBV has not been detected in normal nasopharyngeal epithelial cells adjacent to EBV positive tissue but has been detected in *in situ* nasopharynx carcinomas, which are presumed precursor lesions of NPC development (Pathmanathan et al., 1995; Niedobitek et al., 1996). Latent infection of EBV is the predominant status in nature in NPC, and the default program of EBV infection is lytic replication. The switch from latency to the lytic cycle is known as EBV reactivation, this process will amply the viral genomes to generate abundant viral particles for promoting EBV-associated carcinogenesis by entering into a latent state to remain within the host indefinitely (Murata and Tsurumi, 2014). Thus, the establishment of latent infection represents an important step in the pathogenesis of NPC. Overexpression of cyclin D1 or its associated factors has been reported to enable persistent infection of EBV in latency (Tsang et al., 2012), which may be due to the enhanced glucose metabolism by activated cyclin D1 signaling. Interestingly, Adamson and his colleagues showed that treating the EBV-positive epithelial cells with mTOR inhibitor, rapamycin, could effectively evaluate the lytic replication in a dose- and time-dependent manner, which was indicated by the enhancement of *Zta* and *Rta* transcript and protein levels (Adamson et al., 2014). Since blocking the mTOR activation by rapamycin could cause a starvation status by slowing down the glucose uptake, in other words, EBV prefers to switch into lytic cycle from latency when EBV-positive epithelial cells undergo starvation, this will heighten our knowledge of how the host-viral interactions alteration contributes to the EBV lytic cycle. A better understanding of how glucose metabolism links to EBV lytic reactivation further contributes to NPC pathogenesis that will help in designing new virus-targeted therapies by focusing on lytic cycle combining with metabolism regulation.

### Glucose Metabolism Beneath NPC Escapes From Apoptosis

Apoptosis is a form of cell death required for maintaining the physiological homeostasis of the body. Metabolism has been considered as a driver for carcinogenesis through multiple aspects including promotion of the cancer cells to proliferation and escape from apoptosis (Hanahan and Weinberg, 2011). Inhibition of cell metabolism by glucose deprivation or disturbing the process of glucose metabolism have been extensively considered as approaches to preferentially arrest the carcinogenesis property (Dang, 2012; Hay, 2016; DeBerardinis and Chandel, 2016). Our study showed that EBV-encoded oncoprotein, LMP1, enhanced glucose uptake and aerobic glycolysis; this event is essential for LMP1-mediated carcinogenesis of NPE cells, treatment with glycolytic inhibitor STF-31, or knockdown Glut-1 expression that initiated the apoptotic program as indicated by the increase of cleaved-caspase 3 level, which indicated that aerobic glycolysis would benefit cells against apoptosis (Zhang et al., 2017). By targeting to the FGF1/FGFR signaling with small molecular inhibitors, LMP1-induced cell proliferation also reduced synchronous with the aerobic glycolysis reduction (Lo et al., 2015). LMP1 expression that accelerated the aerobic glycolysis also causes the NPC cells to be insensitive to radiotherapy; Xiao and colleagues reported the exposure of the LMP1-expressed NPC cells into irradiation: the apoptotic level was high if the glycolytic enzyme HK2 knocks down further (Xiao et al., 2014). Similar studies were also observed by Lu et al. (2016) and Jiang et al. (2015) by targeting the DNA-PK/AMPK pathway as well as the TET3/HoxC8 activity. Interestingly, our unpublished data show that induction of EBV lytic cycle by using rapamycin to block the mTOR activation also enhances the cell apoptosis; this event may be explained by mTOR activation–enhanced glycolysis that is essential for maintaining EBV latency infection to escape apoptosis. Taken together, these findings indicated that metabolism in EBV associated cancer may beneath the cells that are insensitive to apoptotic induction, targeting that metabolism may provide new strategy for EBV-infected NPC treatment.

### Glucose Metabolism Alters the Tumor Microenvironment

The tumor microenvironment is known to play an important function for modulation of tumor growth, progression, and metastasis to distant sites, for the development of acquired treatment resistance, and, finally, for poor patient prognosis (Albini and Sporn, 2007). The tumor microenvironment contains all the elements that are required for tumor progression including malignant and immune cells, stroma, fibroblasts, and the vasculature and lymphatics of the tumor. A typical feature of tumor microenvironment is the acidic environment, which plays key roles for carcinogenesis in the tumor progression. As a product of aerobic glycolysis, lactate is the main contributor for acidosis of the cell microenvironment due to the dynamic shuttling of lactate and proton from cancer cells to the extracellular site. Lactate seems to be involved in all the major steps in cancer progression: angiogenesis, immune escape, cell migration, metastasis, and self-sufficiency of cancer cells (Hanahan and Weinberg, 2011; Hirschhaeuser et al., 2011; Dhup et al., 2012; Justus et al., 2013). EBV infection in NPC has been postulated to be associated with NPC pathogenesis by affecting the multiple aspects including angiogenesis (Sakakibara and Tosato, 2009; Jonigk et al., 2014; Yang et al., 2015), cell motility (Dawson et al., 2008; Shair et al., 2008; Wasil and Shair, 2015), and immune escape (Munz and Moormann, 2008; Merlo et al., 2010; Ning, 2011); this seems to be matched with the function of lactate. In cancer, lactate plays an important role in angiogenesis by stimulating vascular endothelial growth factor (VEGF) protein expression (Dhup et al., 2012; Polet and Feron, 2013). LMP1 increases JNKs/c-Jun signaling activation to enhance the HIF-1/VEGF activity and induce angiogenesis. It is interesting that HIF-1 is an important regulator for glucose metabolism by promoting the glycolytic enzymes activation (Denko, 2008), and it could be explained that the angiogenesis property induced by LMP1 in this case may be due to the accumulated lactate in the extracellular site. It has been known for a long time that lactate level is closely correlated with metastasis in different types of cancers; high concentrations of lactate are associated with distant cancer metastasis (Schwickert et al., 1995; Walenta et al., 2000; Walenta and Mueller-Klieser, 2004; Dhup et al., 2012). As an oncogene, LMP1 that drives NPC motility through multiple pathways has been well established, including ERK-MAPK pathway (Dawson et al., 2008), FGD4/CDC42 pathway (Liu HP et al., 2012), and NF-κB/TNFAIP2 (Chen et al., 2014), and it is attractive that all these signaling pathway activations have a common phenotype: regulating glucose metabolism. Based on their correlation, we can hypothesize that LMP1-enhanced lactate production may be involved in this event.

Lactate has been reported to contribute to immune escape from different approaches, like suppressing monocyte migration and release of cytokines (Goetze et al., 2011), inhibiting the activation of T cells and natural killer cell (Fischer et al., 2007; Husain et al., 2013). Acidosis environment could be involved in lactate to suppress immune system (Choi et al., 2013). NPC is characterized by substantial immune infiltrate in the tumor microenvironment including dendritic cells, monocytes, T cells, and B cells. The production of inflammatory cytokines largely seems to be essential for the infiltration of lymphoid NPC progression. Expression of LMP1 in NPC cells recruits T regulatory cells (T_regs_) through NF-κB signaling activation, which distributes around the tumor and contributes to inhibit the activation of innate cytotoxic T-cell responses; the T_regs_ enhances the secretion of the chemokine CCL20 and IL-10 and increases the migration of itself toward the tumor (Marshall et al., 2003; Baumforth et al., 2008), subsequently contributing to play the immune suppressive functions. Similar studies also observed that EBV microRNAs (Nachmani et al., 2009; Haneklaus et al., 2012; Lin et al., 2013) and lytic genes (Morrison and Kenney, 2004; Bristol et al., 2010; Michaud et al., 2010; Shen et al., 2015) also contribute to evade the host immune destruction, for example, the BART-miRNA15-3p could block the production of IL-1 which is a pro-inflammatory cytokine *via* inhibition of the NLRP3 inflammasomal activity (Haneklaus et al., 2012). The EBV lytic gene, BZLF1, was found to inhibit interferon-α (IFNα) production by monocytes through inhibition of JAK/STAT signaling and finally induce a state of type I IFN irresponsiveness (Michaud et al., 2010). It is quite interesting that the above summarized EBV-associated immune response can affect by the lactate and it associated acidosis environment, which suggests to us that aerobic glycolysis–mediated microenvironment alteration may play a role in EBV modulation of host immune responses.

## Targeting EBV-Induced Metabolic Dysregulation as Promising Strategies for NPC Therapy

In a previous clinical-based study, it has been observed that EBV infection was positively correlated with the expression of Glut-1 (Zhou et al., 2017). Compared with the healthy group, the proportion of Glut-1–positive individuals is significantly higher in NPC patients (58.73% versus 29.17%). Furthermore, the clinical stage and the ratio of the lymph node metastasis are likely to be higher but the 3-year survival rate is lower in Glut-1–positive patients, indicating that Glut-1 is a poor prognosis factor in NPC (Zhou et al., 2017). In another study, the expression of HK2 has been positively correlated to LMP1. Overexpression of HK2 in NPC patients was indicated to be a poor prognosis factor in terms of the overall survival (OS) in NPC patients (Xiao et al., 2014). Our study confirmed that highly expressed PDHE1α in NPC tissues is associated with advanced tumor stages and lower OS of the NPC patients (Zhang et al., 2019). Moreover, EBV-miR-BART1 expression in NPC has been reported that it is highly associated with higher clinical stages of the disease (Cai et al., 2015b). Collectively, EBV infection may drive the cell metabolic reprogramming to contribute to the cancer progression. Hence, targeting metabolic dysregulation in EBV-associated cancer therapy has greatly attracted researcher’s attentions these years.

The early stages of NPC patients can be effectively treated by combined chemotherapy and radiotherapy, however, the long-term survival rate of NPC patients is still poor if detected at advanced stages (Cheng et al., 2000; Wei and Sham, 2005). Thus, developing new strategies for NPC clinical treatment is an urgent need. Due to the unique role of EBV in NPC aerobic glycolysis, interfering viral-mediated metabolic reprogramming to revise the cell metabolic behavior has been shown to be promising therapeutic strategies in preclinical studies. For instance, overexpression of LMP1 suppressed gene *HoxC8* in NPC cells to attenuate the function of LMP1 in glycolytic metabolism, which eventually led to the growth inhibition of cells both *in vitro* and *in vivo* (Jiang et al., 2015). Inhibition of LMP1-induced protein FGFR1 in LMP1-expressed epithelial cell lines could suppress cell proliferation, migration, and invasion to decrease the risk of the cell malignant transformation (Lo et al., 2015). Silencing the EBV-miR-BART1-5P significantly decreased the glycolytic activity and propagation of NPC cells (Lyu et al., 2018).

As aforementioned, EBV infection and EBV-encoded oncoproteins activate multiple signaling pathways that regulate NPE cell proliferation and malignant transformation, including PI3K/AKT/mTOR axis, AMPK/mTOR pathway, IKK/NF-κB pathway, and cell cycle pathway. The mTOR plays an irreplaceable function in the governing of nutrient balance and energy metabolism, and it has been shown to be activated in EBV-transformed cells (Moody et al., 2005; Wlodarski et al., 2005; McFadden et al., 2016). As an effective inhibitor of mTORC1, rapamycin or its derivates also have been proved to have inhibitory effect in EBV-associated cancers (Toh et al., 2006; Cen and Longnecker, 2011; Zhang et al., 2017) and reduce the aerobic glycolysis (Zhang et al., 2017). Similarly, targeting the AMPK signaling by metformin and AICAR (Lo et al., 2013; Lu et al., 2016) and modulating the activity of NF-κB signaling by NBD (Zhu et al., 2016b; Zhang et al., 2017) in EBV-associated epithelial cells have attenuated the EBV and its oncoproteins-droved cell growth and malignant transformation, suggesting that disrupting the above mentioned pathways by related inhibitors may provide an additional benefit to antiglycolytic therapies for EBV-infected NPC. The benefits of the combined use of these inhibitors with conventional NPC treatment modalities, namely, radiotherapy, chemotherapy, and the recently initiated immune check point therapy, remain to be explored.

The activity of glucose metabolism is highly increased in cancer cells compared with those in normal cells due to their high demands of energy and macromolecules. The former one metabolized pyruvate to lactate by LDHA and transport out to extracellular space to modulate the microenvironment, whereas the latter one transported pyruvate into trichloroacetic acid cycle in mitochondria. Thus, targeting on dysregulated metabolic enzymes that are preferentially activated in cancer cells will represent promising therapeutic strategies. As discussed above, Glut-1 is regarded as a tumor marker due to its significant clinical relevance in EBV-associated malignancy of NPC (Sommermann et al., 2011; Szablewski, 2013; Zhang et al., 2017; Zhou et al., 2017). Glut-1–specific chemical inhibitors, including STF-31, have shown to have a strong effect to selectively kill NPC cells *in vitro* (Chan et al., 2011; Liu Y et al., 2012; Zhang et al., 2017). Furthermore, inhibition of HK2 increased the radiotherapy sensitivity, which indicated that HK2 could be used as a target to enhance the treatment effects (Xiao et al., 2014). Another key enzyme, PKM2, which regulates the last step of glycolysis, and dephosphorylation of phosphoenolpyruvate to pyruvate, is also highly increased in LMP1-expressed cells (Lo et al., 2015). Taken together, due to their important functions in different stage of glycolysis, targeting activities of glycolytic enzymes may represent a promising approach for NPC treatment by directly interfering with glycolysis process.

To date, although there are no drugs that are targeting aerobic glycolysis available in NPC treatment, development of such kind of drugs may represent to provide a novel therapeutic option for the disease management. Serval EBV-related glycolytic genes could serve as potential targets for the cancer drug development; remarkably, drugs against HIF1α have achieved a great progression. Topotecan and bortezomib are two HIF1α inhibitors that have been approved for the treatment of solid cancers by the US Food and Drug Administration (FDA). Two phase II clinical trials, the NCT00002515 and NCT00305734 trials, have been carried out to evaluate the effectiveness of topotecan in combination with other cancer drugs in treating patients who have advanced rare caners, including NPC, and the effectiveness of bortezomib in combination with gemcitabine in treating patients who have recurrent or metastatic NPC, respectively. Additionally, more HIF1α-targeting drugs are under development. Two phase I trial (NCT00687934 and NCT01183364) to study another HIF1α inhibitor ganetespib alone or in combination with docetaxel in treating solid tumors have been completed, and the positive result collected from NCT00687934 has been published (Goldman et al., 2013). These studies have inspired investigators to develop highly effective therapeutic methods through targeting EBV-induced metabolic reprogramming in NPC.

## Concluding Remarks

The role of EBV infection in NPC pathogenesis has been enigmatic. The underlying mechanism that supports latent EBV infection and tumorigenesis in NPC is yet not fully defined. An interactive interplay between glucose metabolism reprogrammed by EBV infection and its encoded-oncogene expression may modulate malignant properties of NPC cells including angiogenesis, invasion, metastasis, and resistance to therapy. Considering the prevalent impact of glucose metabolism in NPC malignances, an in-depth understanding of the mechanisms of EBV-regulated metabolic dynamics may aid the development of functional therapeutic application in modulating metabolic rewiring.

More efforts in the future are needed to identify key molecular determinants that are involving in EBV-reprogrammed glucose metabolism in order to provide relevant insights into queries like the following: how does EBV-reprogrammed glucose metabolism contribute to EBV latent infection? Does glucose metabolism contribute to EBV latent and lytic switching? How to suppress NPC metastasis and potentially reverse resistance to clinical treatment in NPC patients by targeting metabolic process?

## Author Contributions

TY, CY, and SM draft the manuscript; ZL, WA, and JZ discussed the review content; WA and JZ modified the manuscript. All authors contributed to the article and approved the submitted version.

## Funding

This project was supported by funding from the National Natural Science Foundation of China (82002986) and Guangdong Basic and Applied Basic Research Foundation (2019A1515110041, 2021A1515011126).

## Conflict of Interest

The authors declare that the research was conducted in the absence of any commercial or financial relationships that could be construed as a potential conflict of interest.

## Publisher’s Note

All claims expressed in this article are solely those of the authors and do not necessarily represent those of their affiliated organizations, or those of the publisher, the editors and the reviewers. Any product that may be evaluated in this article, or claim that may be made by its manufacturer, is not guaranteed or endorsed by the publisher.
